# Variable Phenotypic Manifestations of Novel Compound Heterozygous CRPPA Variants in Two Iranian Siblings With Limb‐Girdle Muscular Dystrophy

**DOI:** 10.1002/mgg3.70312

**Published:** 2026-10-22

**Authors:** Parnian Alagha, Fatemeh Ahangari, Ariana Kariminejad, Aida Ghasemi, Soheila Gholami, Neda Sadatian, Roxana Kariminejad, Kimia Kahrizi, Ali Asghar Okhovat, Hossein Najmabadi

**Affiliations:** ^1^ Kariminejad ‐ Najmabadi Pathology & Genetics Center Tehran Iran; ^2^ Genetics Research Center University of Social Welfare and Rehabilitation Sciences Tehran Iran; ^3^ Neuromuscular Research Center Tehran University of Medical Sciences Tehran Iran

**Keywords:** array‐CGH, copy number variant (CNV), *CRPPA*, dystroglycanopathies, limb‐girdle muscular dystrophy (LGMD), whole exome sequencing (WES)

## Abstract

**Background:**

Dystroglycanopathies are a clinically and genetically heterogeneous group of muscular dystrophies caused by impaired glycosylation of *α*‐dystroglycan, with variants in multiple genes, including *CRPPA*, which is associated with a broad spectrum ranging from congenital muscular dystrophy to limb‐girdle muscular dystrophy.

**Method:**

The proband underwent whole exome sequencing, while Sanger sequencing was used for segregation analysis and oligonucleotide array‐CGH was performed to confirm the copy number variant.

**Results:**

We report two siblings harboring an identical novel compound heterozygous *CRPPA* genotype, comprising a novel c.267G>C missense variant and a deletion encompassing exons 9–10. Despite sharing the same genotype, the siblings showed marked phenotypic variability. The female sibling developed childhood‐onset disease with proximal weakness involving both the upper and lower limbs, whereas the male sibling presented with late‐onset disease and weakness confined to the proximal lower limbs, accompanied by markedly discordant serum creatine kinase levels (1193 vs. 460 U/L).

**Conclusion:**

These findings expand the mutational and clinical spectrum of CRPPA‐related dystroglycanopathy, highlight marked intrafamilial phenotypic variability, and emphasize the importance of integrated single‐nucleotide and copy number variant analysis in the genetic diagnosis of neuromuscular disorders.

## Graphical abstract

A novel compound heterozygous CRPPA genotype (missense variant and exon 9‐10 deletion) identified in two siblings resulted in marked intrafamilial phenotype variability, ranging from childhood‐onset to late‐onset muscular dystrophy, highlighting the importance of combined SNV and CNV analysis.

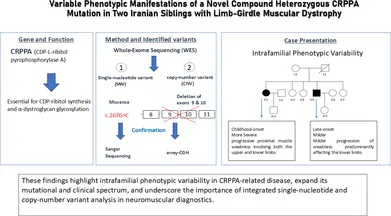

## 1 Introduction

Dystroglycanopathy represents a subgroup of muscular dystrophies caused by defective glycosylation of *α*‐dystroglycan (*α*‐DG) and is characterized by marked clinical and genetic heterogeneity (Song et al. 2020, 2021). To date, at least 19 causative genes have been identified (Song et al. 2020), reflecting the complexity of the *α*‐dystroglycan glycosylation pathway. One of these genes is *CRPPA* (formerly known as *ISPD*), which encodes CDP‐L‐ribitol pyrophosphorylase A, an enzyme responsible for synthesizing CDP‐ribitol, a critical substrate required for subsequent ribitol phosphate modification of *α*‐dystroglycan by downstream glycosyltransferases (Kanagawa 2021).

The phenotypic manifestations of variants in the *CRPPA* gene range from severe disorders, such as congenital muscular dystrophy (CMD), to milder forms, such as limb‐girdle muscular dystrophy (LGMD) (Kanagawa 2021; Cirak et al. 2013). LGMDs exhibit a clinically diverse group of disorders with an estimated prevalence of about 2 per 100,000 people and are characterized by progressive proximal weakness and dystrophic features on muscle biopsy. LGMDs typically manifest later in life, during late childhood, adolescence, or adulthood (Georganopoulou et al. 2021). Accordingly, the majority of reported individuals with CRPPA‐related disease manifest symptoms in infancy or early childhood, often accompanied by additional systemic or neurodevelopmental involvement. Importantly, although biallelic single‐nucleotide variants (SNVs) in *CRPPA* are well documented, only a few reports describe compound heterozygous genotypes combining SNVs and copy number variants (CNVs), and the phenotypic consequences of such variants remain incompletely understood.

In this study, we describe a family with two siblings carrying the same novel compound‐heterozygous *CRPPA* genotype, comprising a missense variant and a CNV. The siblings exhibited marked differences in age of onset and clinical severity, demonstrating intrafamilial phenotypic variability. This case underscores the importance of combined SNV and CNV analysis for accurate diagnosis of heterogeneous neuromuscular disorders and expands the mutational and clinical spectrum of *CRPPA*‐related disease.

## 2 Materials and Methods

### 2.1 Genetic Analysis

The proband (Individual II.5) underwent whole exome sequencing (WES) at the Kariminejad ‐ Najmabadi Pathology & Genetics Center. Genomic DNA was extracted from peripheral blood cells using the standard salting‐out protocol. Target enrichment was performed using the Twist Exome V2.0 kit (Twist Bioscience, South San Francisco, CA, USA), followed by sequencing of the enriched exonic regions on the Illumina NovaSeq 6000 platform (Illumina, San Diego, CA, USA), in accordance with the manufacturer's instructions. Raw sequencing reads were aligned to the human reference genome assembly GRCh38 (hg38) by Illumina DRAGEN Bio‐IT Platform v3.9 (Behera et al. 2025). Variants were annotated using VarSeq v2.6.0 (Golden Helix, Bozeman, MT, USA) and the AnnotSV tool v3.4.4 (Geoffroy et al. 2018). Variant classification according to the American College of Medical Genetics and Genomics (ACMG) (Richards et al. 2015) guidelines was performed using VarSome (Kopanos et al. 2019) and Franklin (Genoox) (https://franklin.genoox.com).

Segregation of the single‐nucleotide variant was assessed by Sanger sequencing for the proband and eight additional family members using an ABI 3130 Sequencer (Life Technologies, Carlsbad, CA, USA). Sequence reads were aligned to the reference genome using CodonCode Aligner software (version 8.0.1). To confirm the copy number variant (CNV) and evaluate segregation, whole‐genome oligonucleotide array comparative genomic hybridization (array‐CGH) was performed in the proband, the affected sibling (II.1), and the mother (I.1), using the SurePrintG3 ISCA v2 8 × 60K platform (Agilent Technologies, SantaClara, CA, USA). Data were analyzed using Agilent Cytogenomic software version 4.

## 3 Results

### 3.1 Case Report

The proband (Individual II.5) was a 42‐year‐old male born to non‐consanguineous parents (Figure 1), who presented with progressive proximal lower limb muscle weakness that began at the age of 41, manifested primarily as difficulty climbing stairs. Neurological examination revealed proximal muscle weakness predominantly affecting the lower limbs, without evidence of calf hypertrophy, scapular winging, or joint hypermobility. Serum creatine kinase (CK) level was elevated at 1193 U/L (Normal value: 24–195 U/L). Electromyography (EMG) demonstrated features consistent with chronic non‐irritable myopathy. No ocular or central nervous system involvement was observed, and cognitive function was normal. His affected sister (Individual II.1), a 52‐year‐old female, had a markedly different clinical course despite carrying the same compound heterozygous CRPPA genotype. She developed symptoms at approximately 10 years of age, with progressive proximal muscle weakness involving both the upper and lower limbs. Laboratory evaluation revealed a mildly elevated serum CK level of 460 U/L. Muscle biopsy revealed dystrophic changes consistent with a myopathic process. EMG findings were comparable to those observed in the proband (Individual II.5), demonstrating chronic non‐irritable myopathy. There was no evidence of ocular or central nervous system involvement, and cognitive function was normal.

**FIGURE 1:**
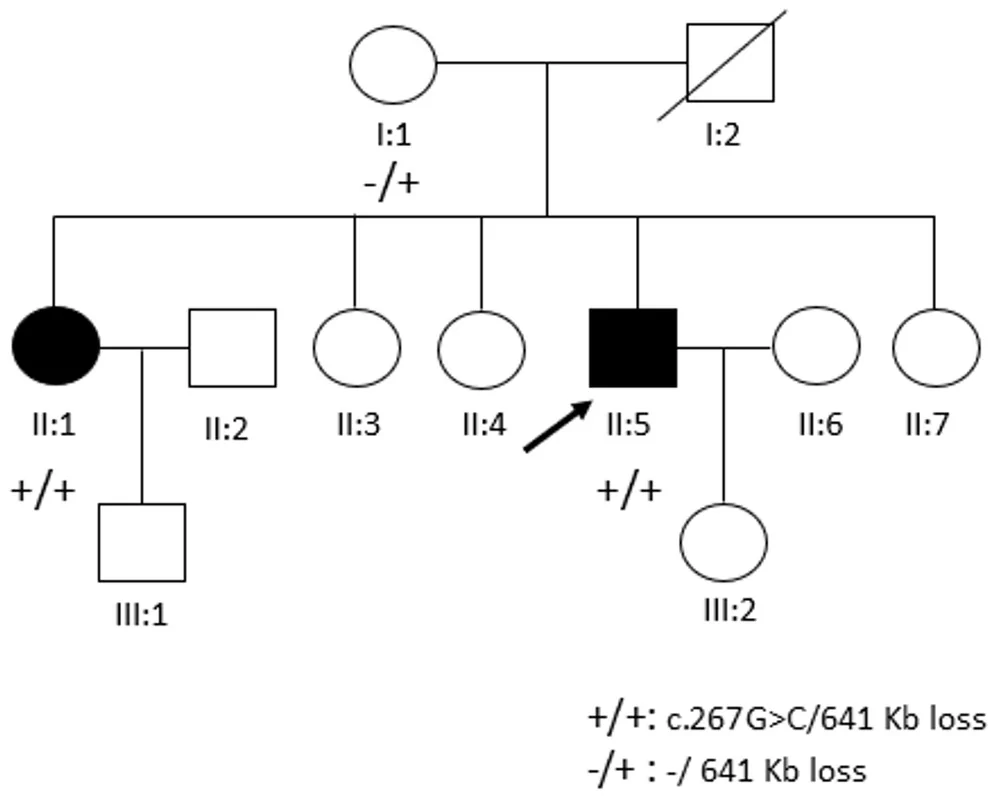
Family pedigree. Individual II.5 is the proband (arrow marked). Individual II.1 was affected and carried both CRPPA variants. The segregation results of the unaffected siblings and other tested relatives are shown in Figure S1. Square: Male; circle: Female; white‐marked: Unaffected; black‐marked: Affected; slash‐marked: Deceased.

WES identified two heterozygous variants in the *CRPPA* gene: a missense single‐nucleotide variant, c.267G>C (p.Trp89Cys), and a heterozygous copy number variant defined as NC_000007.14: g.(?_15559642)_(16216697_?)del. The missense variant was absent from both the Genome Aggregation Database v4.1 (Genome Aggregation Database, https://gnomad.broadinstitute.org/) and the Iranian genomic population database (Iranome n.d.; Fattahi et al. 2019). Prediction tools (PolyPhen‐2, MutationTaster, CADD v1.6 (Rentzsch et al. 2019), FATHMM, REVEL, PrimateAI) consistently suggested a deleterious effect and the pathogenicity of this variant. Based on the available evidence, the variant was classified as a variant of uncertain significance (VUS) according to ACMG/AMP recommendations (PM2, PP3, PP1).

The CNV constitutes a gross loss of 641 Kb of 7p21.2p21.2, encompassing exons 9 and 10 of the *CRPPA* gene (OMIM *614631), as well as additional genes including MEOX2, LOC105375166, MEOX2‐AS1, and ISPD‐AS1. This deletion has not been previously reported and is also absent from the Database of Genomic Variants (DGV) (DGV, n.d.). Based on its size, gene content, and segregation data, the CNV was classified as likely pathogenic.

Structural modeling using AlphaFold2 localized Trp89 to a highly conserved region within the N‐terminal catalytic domain immediately adjacent to a β‐strand, with high prediction confidence (pLDDT > 90) (Figure 2A,B). HOPE (Venselaar et al. 2010) server predicted that substitution of the bulky tryptophan residue with the smaller cysteine would create a space within the protein core, potentially reducing local structural stability. Consistent with these structural observations, multiple *insilico* prediction tools (PolyPhen‐2, MutationTaster, REVEL, FATHMM, CADD, PrimateAI) uniformly supported a deleterious effect of the variant (Table 1).

**FIGURE 2:**
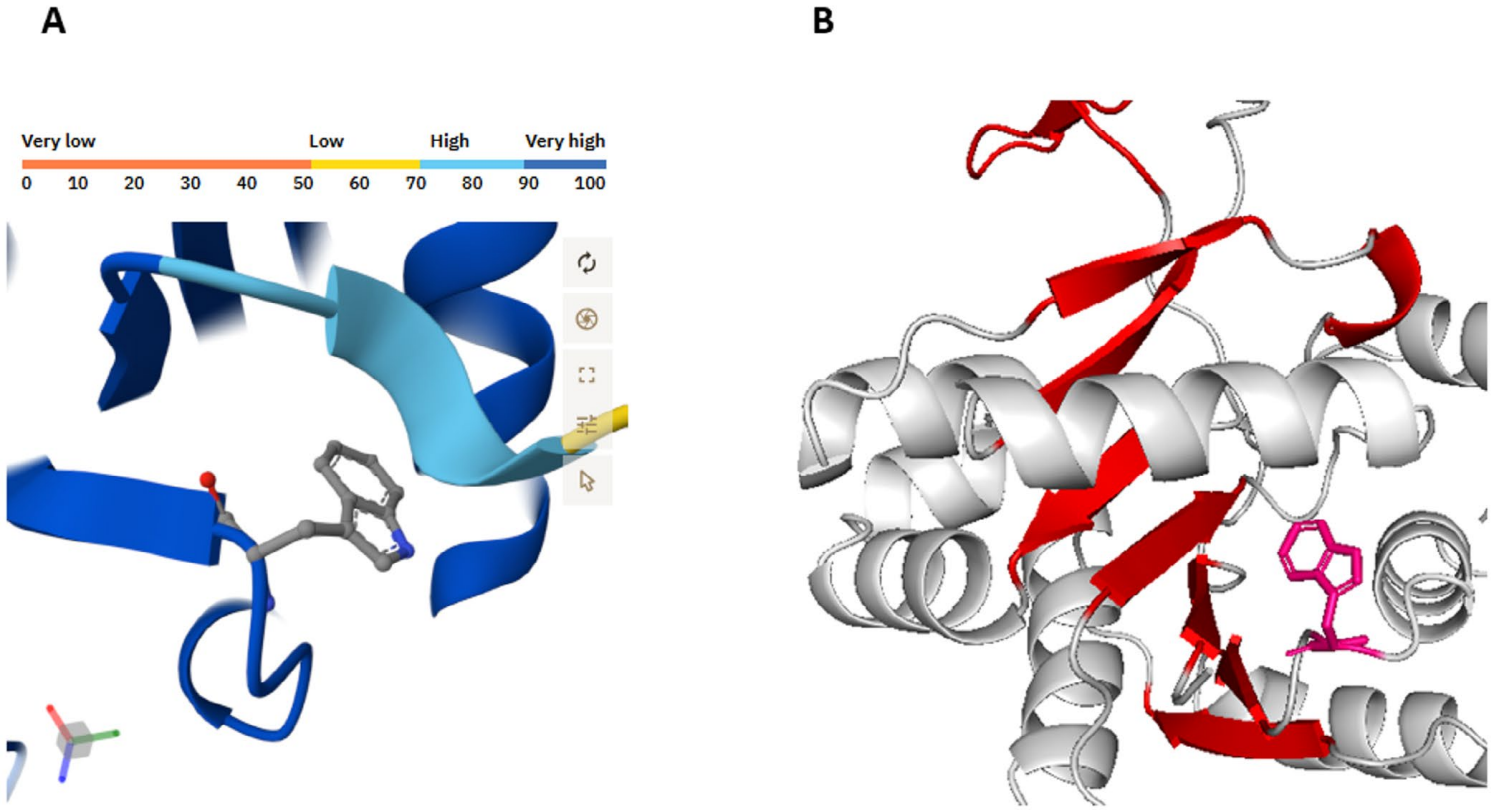
(A) AlphaFold2 structural model of CRPPA showing the predicted position of residue Trp89. The model is colored by per‐residue confidence score (pLDDT): Very high (dark blue, > 90), high (light blue, 70–90), low (yellow, 50–70), and very low (orange, < 50). Residue Trp89 (shown as gray sticks) is located within a region of very high predicted confidence (pLDDT > 90), (B) Close‐up view of Trp89 located immediately adjacent to a β‐strand (red).

**TABLE 1:** In silico prediction scores for the novel CRPPA c.267G>C (p.Trp89Cys) variant.

In silico tool	Prediction
CADD	Deleterious (29.7)
REVEL	Deleterious (supporting 0.75)
PolyPhen‐2	Possibly damaging
MutationTaster	Disease causing/damaging
FATHMM	damaging
PrimateAI	Deleterious (moderate 0.88)

Segregation analysis demonstrated the presence of both variants in the affected sister (Individual II.1) (Figures 1, 3 and S1). The c.267G>C missense variant was absent in the mother (Individual I.1), while the oligonucleotide array‐CGH confirmed the presence of the 641‐kb deletion in the mother. These findings are consistent with a compound heterozygous state in the affected siblings.

**FIGURE 3:**
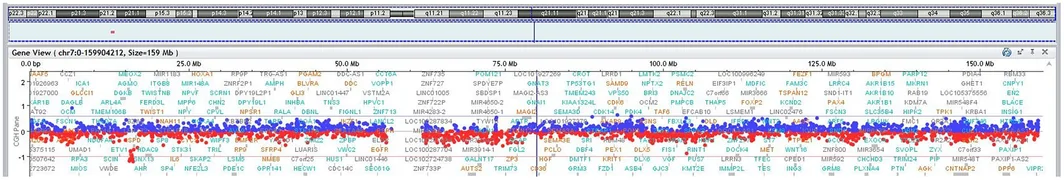
Array comparative genomic hybridization (array‐CGH) analysis demonstrating a heterozygous copy number loss at chromosome 7p21.2 (approximately 641 kb), encompassing exons 9–10 of the CRPPA gene. Genomic coordinates are shown based on GRCh38.

Despite sharing the same compound heterozygous *CRPPA* genotype, the siblings showed marked phenotypic differences. The female sibling developed symptoms approximately three decades earlier than the male sibling (10 vs. 41 years), exhibited proximal weakness involving both the upper and lower limbs, and had a lower serum CK level (460 vs. 1193 U/L). The initial presentation also differed, with the male proband presenting with difficulty climbing stairs, whereas the female sibling initially presented with generalized proximal muscle weakness. In contrast, the male sibling presented with weakness restricted to the proximal lower limbs. In addition, muscle biopsy showing dystrophic changes was available only for the female sibling. Both siblings shared chronic non‐irritable myopathy on EMG, normal cognitive function, and the absence of ocular or central nervous system involvement.

The proband and affected sister (Individual II.1) received supportive metabolic therapy, including coenzyme Q10 (100 mg daily), riboflavin (100 mg twice daily), and L‐carnitine (100 mg daily). At follow‐up, the proband demonstrated only mild proximal weakness of the lower limb muscles.

## 4 Discussion

In dystroglycanopathies, abnormal glycosylation of dystroglycan (DG) impairs ligand binding and basement membrane interactions, reducing muscle membrane stability. CRPPA catalyzes the synthesis of cytidine diphosphate‐ribitol (CDP‐ribitol), a critical step in DG glycosylation. Disruption of this enzymatic step impairs DG glycosylation and contributes to the pathogenesis of dystroglycanopathies (Kanagawa 2021).

In this study, we identified a novel compound heterozygous *CRPPA* genotype in two siblings with limb‐girdle muscular dystrophy. Notably, despite an identical genotype, the siblings differed markedly in age at onset, muscle weakness distribution, and CK levels. Together, these findings demonstrate marked intrafamilial phenotypic variability within a single *CRPPA*‐affected family.

The identified variants comprise a missense SNV, c.267G>C (p.Trp89Cys), located in exon 2 within the N‐terminal catalytic domain, and a CNV encompassing exons 9–10 in the C‐terminal region. The c.267G>C variant has not been previously reported and affects the N‐terminal catalytic domain, where missense variants may disrupt active‐site integrity (Riemersma et al. 2015). Meanwhile, the C‐terminal domain of *CRPPA* contains multiple truncating variants, including frameshift variants and exon‐level deletions, that have been reported to cause disease by altering cytidyltransferase activity (Riemersma et al. 2015; Zhang et al. 2025), and are associated with remarkable phenotypic variability. To place our findings in context, we reviewed published studies (2012–2026) reporting *CRPPA* variants and associated clinical data (Table S1). The distribution of published *CRPPA* variants across the protein and their associated clinical phenotypes is summarized in Figure 4. Although the Human Gene Mutation Database (HGMD Professional 2024.4) (HGMD n.d.) currently lists 54 disease‐associated *CRPPA* variants, with missense variants representing the predominant class and gross deletions constituting a smaller proportion, our analysis was restricted to published cases with sufficient clinical information to allow genotype–phenotype comparison. Among 59 patients, the majority, 37 patients (62.7%), were compound heterozygous, while a smaller subset of 20 (33.9%) carried homozygous variants. In two patients (3.4%) only a single identifiable *CRPPA* allele was reported. One of these two patients also carried a variant in a second gene (*POMT1*), which was excluded from allele analysis. Notably, pathogenic variants were distributed throughout the coding region; exon 9 emerged as a recurrent site of variation, highlighting its functional importance.

**FIGURE 4:**
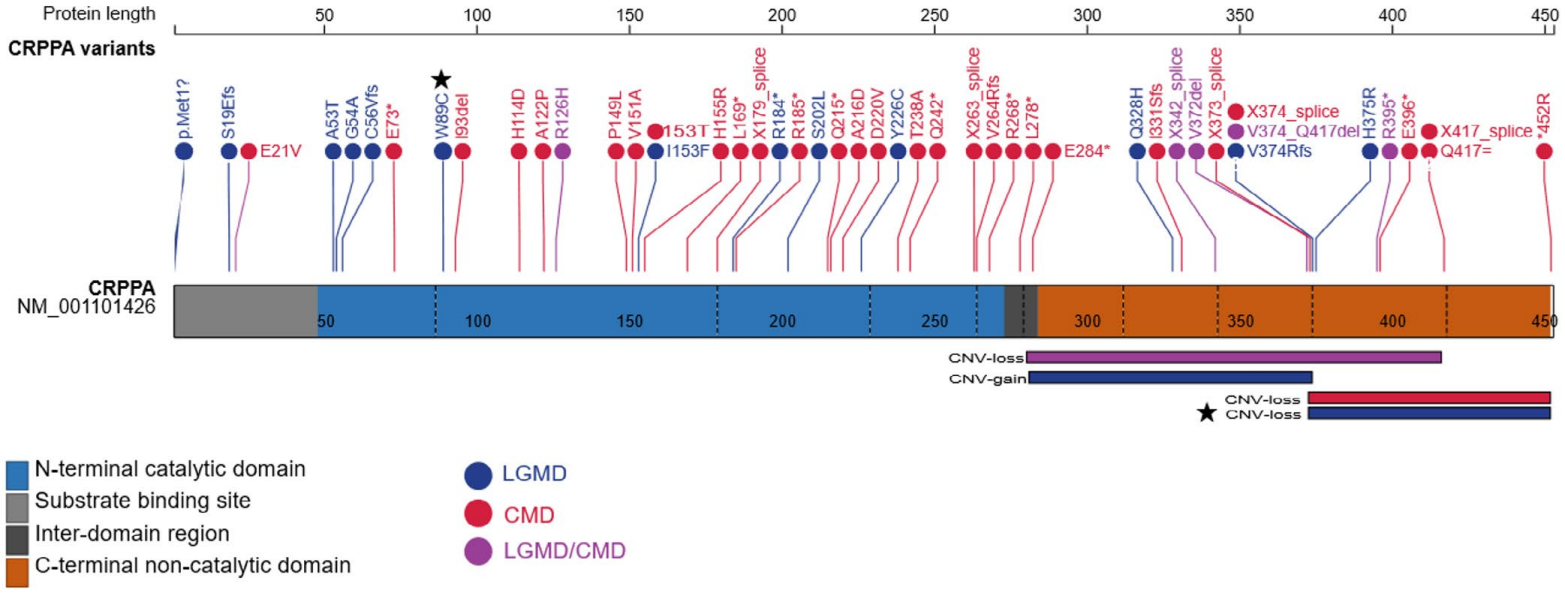
Distribution of published *CRPPA* variants across the protein and their associated clinical phenotypes. Variants identified in the literature review (2012–2026) are mapped according to their position within the *CRPPA* protein. Variants are color‐coded by the reported clinical phenotype (LGMD, CMD, or LGMD/CMD). CMD includes congenital muscular dystrophy and related severe dystroglycanopathy phenotypes, including Walker–Warburg syndrome and Muscle‐Eye‐Brain disease. Functional domains of *CRPPA* are indicated below the protein schematic. The novel SNV and the CNV identified in the present study are marked with an asterisk (*). The figure was initially generated using ProteinPaint (https://proteinpaint.stjude.org/) (Zhou et al. 2016) and then edited in Adobe Illustrator.

Reported deletions most frequently involve exon 9 and, less commonly, extend to exon 10, occurring in both homozygous and predominantly compound heterozygous states (Table S1). Most published cases carrying these deletions presented with severe congenital dystroglycanopathy accompanied by cerebellar hypoplasia, ocular abnormalities, and developmental delay (Song et al. 2020; Roscioli et al. 2012; Willer et al. 2012; Luo et al. 2025). However, milder phenotypes have also been reported, particularly when exon‐level deletions occur in compound heterozygosity with missense variants. Notably, Luo et al. (2025) described a patient carrying a deletion involving exons 6–9 who presented with LGMD phenotype accompanied by cerebellar and brainstem hypoplasia and cardiac involvement. Although genotype–phenotype correlations remain incompletely defined, previous studies indicate that specific compound heterozygous genotypes, particularly those involving frameshift or truncating variants, may lead to early‐onset severe phenotypes, whereas other variant combinations can result in later‐onset, milder limb‐girdle presentations (Luo et al. 2025). This variability is likely related to differing degrees of residual protein function. In the present family, the combination of the novel p.Trp89Cys variant and the exon 9–10 deletion is consistent with, although does not prove, a hypomorphic disease mechanism. The structural predictors provide additional support for the predicted pathogenicity of p.Trp89Cys. Although AlphaFold2 localized the variant to a highly conserved region of the N‐terminal catalytic domain with high prediction confidence, HOPE analysis suggested that substitution of tryptophan by cysteine could create an empty space within the protein core and potentially alter structurally important domain contacts. Although these findings support a deleterious effect, functional studies are required to confirm their biological significance.

Despite marked phenotypic heterogeneity, individuals with *CRPPA*‐related disease who present with a limb‐girdle muscular dystrophy phenotype often share core neuromuscular features, including progressive proximal muscle weakness, gait difficulties, and elevated serum creatine kinase levels. Disease onset is most commonly reported in infancy or early childhood, particularly among patients with biallelic truncating or exon‐level deletions, although later‐onset presentations have also been described (Table S1). Additional neurological manifestations, such as cerebellar and brainstem hypoplasia, may be observed in a subset of patients, underscoring the broad clinical spectrum of *CRPPA*‐associated dystroglycanopathy. The age at onset of 41 years in the male proband is markedly later than that reported in most *CRPPA*‐related LGMD cases. As most reported cases are childhood‐onset and severe, milder adult‐onset presentations may be underrecognized. This finding broadens the known age‐of‐onset spectrum of *CRPPA*‐related LGMD.

In summary, this family demonstrates marked intrafamilial phenotypic variability in *CRPPA*‐related dystroglycanopathy, with childhood and late‐onset presentations arising from the same compound heterozygous genotype. Our findings broaden the recognized clinical spectrum of *CRPPA*‐associated disease and underscore the complexity of genotype–phenotype relationships. Importantly, this diagnosis should be considered even in adult‐onset proximal muscle weakness without the brain or eye abnormalities typically associated with more severe phenotypes. A comprehensive genetic evaluation, including the assessment of both single‐nucleotide and copy number variants, is essential for establishing an accurate diagnosis of heterogeneous neuromuscular disorders.

## Author Contributions

**Parnian Alagha:** formal analysis, visualization, credit taxonomy, writing – original draft. **Fatemeh Ahangari:** investigation, methodology, validation. **Ariana Kariminejad:** investigation, data curation, clinical evaluation and interpretation. **Aida Ghasemi:** investigation. **Soheila Gholami:** investigation. **Neda Sadatian:** investigation. **Roxana Kariminejad:** investigation, validation. **Kimia Kahrizi:** writing – review and editing, investigation, data curation, clinical evaluation and interpretation. **Ali Asghar Okhovat:** investigation, data curation, supervision, clinical evaluation and interpretation. **Hossein Najmabadi:** writing – review and editing, funding acquisition, supervision, conceptualization.

## Funding

This study was supported by the University of Social Welfare and Rehabilitation Sciences, Tehran, Iran and Kariminejad ‐ Najmabadi Pathology & Genetics Center, Tehran, Iran (1404/801/A/6/10220).

## Ethics Statement

This study is in accordance with the Helsinki Declaration and has received approval from the Ethics Committee of the University of Social Welfare and Rehabilitation Sciences, Tehran, Iran (IR.USWR.REC.1404.081). Written informed consent was obtained from all participants involved in the study.

## Conflicts of Interest

The authors declare no conflicts of interest.

## Supporting information


**Figure S1:** mgg370312‐sup‐0001‐FigureS1.tif.

**Table S1:** Clinical and molecular characteristics of patients with CRPPA variants reported in the present study and previously published cases.

## Data Availability

The data that support the findings of this study are available from the corresponding author upon request.

## References

[mgg370312-bib-0001] Behera, S. , S. Catreux , M. Rossi , et al. 2025. “Comprehensive Genome Analysis and Variant Detection at Scale Using DRAGEN.” Nature Biotechnology 43, no. 7: 1177–1191. 10.1038/s41587-024-02382-1 PMC12022141 39455800

[mgg370312-bib-0002] Cirak, S. , A. R. Foley , R. Herrmann , et al. 2013. “ISPD Gene Mutations Are a Common Cause of Congenital and Limb‐Girdle Muscular Dystrophies.” Brain 136, no. Pt 1: 269–281. 10.1093/brain/aws312 PMC3562076 23288328

[mgg370312-bib-0003] DGV . n.d. “Database of Genomic Variants (DGV).” https://dgv.tcag.ca/.

[mgg370312-bib-0401] Fattahi, Z. , M. Beheshtian , M. Mohseni , et al. 2019. “Iranome: A Catalog of Genomic Variations in the Iranian Population.” Hum Mutat 40, no. 11: 1968–1984. 10.1002/humu.23880. 31343797

[mgg370312-bib-0004] Geoffroy, V. , Y. Herenger , A. Kress , et al. 2018. “AnnotSV: An Integrated Tool for Structural Variations Annotation.” Bioinformatics 34, no. 20: 3572–3574. 10.1093/bioinformatics/bty304 29669011

[mgg370312-bib-0005] Georganopoulou, D. G. , V. G. Moisiadis , F. A. Malik , et al. 2021. “A Journey With LGMD: From Protein Abnormalities to Patient Impact.” Protein Journal 40, no. 4: 466–488. 10.1007/s10930-021-10006-9 PMC8190568 34110586

[mgg370312-bib-0006] HGMD . n.d. “The Human Gene Mutation Database.” https://www.hgmd.cf.ac.uk/.

[mgg370312-bib-0007] Iranome . n.d. “Iranome Database.” http://iranome.ir/.

[mgg370312-bib-0008] Kanagawa, M. 2021. “Dystroglycanopathy: From Elucidation of Molecular and Pathological Mechanisms to Development of Treatment Methods.” International Journal of Molecular Sciences 22, no. 23: 13162. 10.3390/ijms222313162 PMC8658603 34884967

[mgg370312-bib-0009] Kopanos, C. , V. Tsiolkas , A. Kouris , et al. 2019. “VarSome: The Human Genomic Variant Search Engine.” Bioinformatics 35, no. 11: 1978–1980. 10.1093/bioinformatics/bty897 PMC6546127 30376034

[mgg370312-bib-0010] Luo, J. , Y. Liu , D. Song , et al. 2025. “CRPPA Exon 6–9 Deletion as a Founder Mutation in Chinese Patients With Dystroglycanopathy.” Pediatric Investigation 10: 25–37. 10.1002/ped4.70029 PMC12921627 41726576

[mgg370312-bib-0011] Rentzsch, P. , D. Witten , G. M. Cooper , J. Shendure , and M. Kircher . 2019. “CADD: Predicting the Deleteriousness of Variants Throughout the Human Genome.” Nucleic Acids Research 47, no. D1: D886–D894. 10.1093/nar/gky1016 PMC6323892 30371827

[mgg370312-bib-0012] Richards, S. , N. Aziz , S. Bale , et al. 2015. “Standards and Guidelines for the Interpretation of Sequence Variants: A Joint Consensus Recommendation of the American College of Medical Genetics and Genomics and the Association for Molecular Pathology.” Genetics in Medicine 17, no. 5: 405–423. 10.1038/gim.2015.30 PMC4544753 25741868

[mgg370312-bib-0013] Riemersma, M. , D. S. Froese , W. van Tol , et al. 2015. “Human ISPD Is a Cytidyltransferase Required for Dystroglycan O‐Mannosylation.” Chemistry & Biology 22, no. 12: 1643–1652. 10.1016/j.chembiol.2015.10.014 26687144

[mgg370312-bib-0014] Roscioli, T. , E. J. Kamsteeg , K. Buysse , et al. 2012. “Mutations in ISPD Cause Walker‐Warburg Syndrome and Defective Glycosylation of α‐Dystroglycan.” Nature Genetics 44, no. 5: 581–585. 10.1038/ng.2253 PMC3378661 22522421

[mgg370312-bib-0015] Song, D. , Y. Dai , X. Chen , et al. 2021. “Genetic Variations and Clinical Spectrum of Dystroglycanopathy in a Large Cohort of Chinese Patients.” Clinical Genetics 99, no. 3: 384–395. 10.1111/cge.13886 33200426

[mgg370312-bib-0016] Song, D. , X. Fu , L. Ge , et al. 2020. “A Splice Site Mutation c. 1251G> A of ISPD Gene Is a Common Cause of Congenital Muscular Dystrophy in Chinese Patients.” Clinical Genetics 97, no. 5: 789. 10.1111/cge.13695 31909476

[mgg370312-bib-0017] Venselaar, H. , T. A. Te Beek , R. K. Kuipers , M. L. Hekkelman , and G. Vriend . 2010. “Protein Structure Analysis of Mutations Causing Inheritable Diseases. An e‐Science Approach With Life Scientist Friendly Interfaces.” BMC Bioinformatics 11, no. 1: 548. 10.1186/1471-2105-11-548 PMC2992548 21059217

[mgg370312-bib-0018] Willer, T. , H. Lee , M. Lommel , et al. 2012. “ISPD Loss‐Of‐Function Mutations Disrupt Dystroglycan O‐Mannosylation and Cause Walker‐Warburg Syndrome.” Nature Genetics 44, no. 5: 575–580. 10.1038/ng.2252 PMC3371168 22522420

[mgg370312-bib-0019] Zhang, S. , M. Wu , X. Li , et al. 2025. “A Child of Congenital Muscular Dystrophy‐Dystroglycanopathy With a Novel Variant in the CRPPA Gene: A Case Report and Literature Review.” Transl Pediatr 14, no. 7: 1691–1699. 10.21037/tp-2025-6 PMC12336902 40800181

[mgg370312-bib-0402] Zhou, X. , M. N. Edmonson , M. R. Wilkinson , et al. 2016. “Exploring Genomic Alteration in Pediatric Cancer using ProteinPaint.” Nature Genetics 48, no. 1: 4–6. 10.1038/ng.3466 PMC4892362 26711108

